# Societal participation in the development of orphan drugs: a systematic review

**DOI:** 10.3389/fmed.2025.1653304

**Published:** 2025-09-10

**Authors:** Sanae Akodad, Delphine De Smedt, Hilde Stevens

**Affiliations:** ^1^Institute for Interdisciplinary Innovation in Healthcare (I3h), Faculté de Médecine, Solvay Brussels School of Economics and Management, Université Libre de Bruxelles (ULB), Brussels, Belgium; ^2^Department of Public Health and Primary Care, Ghent University, Ghent, Belgium

**Keywords:** orphan drugs, rare diseases, patient advocacy groups (PAGs), societal participation, clinical trial design, regulatory science, health technology assessment (HTA)

## Abstract

The development of orphan drugs (ODs) remains constrained by structural barriers, including limited scientific knowledge, high clinical uncertainty, and fragile commercial incentives. In parallel, an alternative ecosystem has emerged in which non-commercial societal actors (patients, advocacy groups, and philanthropic entities) contribute to the design, evaluation, and dissemination of therapies for rare diseases. This systematic review provides the first structured synthesis of empirical evidence on societal participation in OD development, with a specific focus on neuromuscular and neurodegenerative disorders. Twenty-one peer-reviewed studies were included, spanning a range of designs from case reports to stakeholder surveys. A functional typology of societal roles was developed, identifying four primary modalities*: Initiator, Accelerator, Translator, and Monitor*. Societal actors contributed to early-stage funding, trial co-design, regulatory engagement, dissemination, and patient preference studies. Their involvement was associated with accelerated development timelines, improved recruitment and retention, enhanced endpoint relevance, increased public literacy, and the emergence of new ethical standards. However, critical tensions remain. The review identifies persistent gaps in representation, risks of tokenism and instrumentalization, blurred governance boundaries, and a lack of longitudinal evaluation frameworks. Although initiatives like EUPATI and IMI PREFER have begun addressing these challenges, further work is required to ensure inclusive, evidence-based, and structurally supported societal engagement. As the European Union Health Technology Assessment Regulation (EU HTAR) reshapes decision-making frameworks, societal participation is no longer peripheral but integral to regulatory legitimacy and therapeutic relevance. Realizing its full potential requires moving beyond anecdotal engagement toward durable, transparent, and evaluable participatory models.

## 1 Introduction

The development of orphan drugs (ODs) for rare diseases has long been hindered by interrelated structural barriers, encompassing scientific, clinical, regulatory, and economic constraints. These include small and geographically dispersed patient populations, limited natural history data, methodological uncertainties in trial design, and prohibitively high development costs that discourage private sector investment (1, 2). While regulatory frameworks such as the *Orphan Drug Act (1983)* in the United States and *Regulation (EC) No 141/2000* in Europe have introduced targeted incentives to stimulate innovation (including market exclusivity, fee waivers, and protocol assistance), these mechanisms presuppose the existence of a viable therapeutic candidate. Yet for over 90% of rare diseases, no such candidate exists: the underlying pathophysiological mechanisms remain poorly understood, and no molecular targets or lead compounds have been identified to initiate a drug development trajectory (3, 4).

In response to these persistent limitations, an alternative ecosystem has gradually emerged in which non-commercial societal actors contribute to multiple phases of therapeutic innovation. These include patient-led initiatives, advocacy coalitions, individual families, and philanthropic or public institutions, which increasingly engage in upstream research, co-design of clinical trials, regulatory dialog, and post-approval dissemination or access efforts. Emblematic cases across regions illustrate the catalytic role of societal actors: in Europe, AFM-Téléthon’s support enabled clinical testing of Olesoxime for spinal muscular atrophy, the AKU Society drove the repurposing of nitisinone for alkaptonuria, and early family–academic collaboration in Belgium laid the groundwork for fenfluramine in Dravet syndrome; in the United States, venture philanthropy by the Cystic Fibrosis Foundation was decisive for ivacaftor, while advocacy groups such as Parent Project Muscular Dystrophy co-authored regulatory guidance with the FDA. While those cases illustrate the catalytic role these actors can play, this broader shift reflects evolving models of biomedical governance that promote more inclusive, participatory, and responsive approaches to innovation (5–7).

The forms taken by such engagement are diverse and evolving. They range from direct financial support of preclinical studies and co-development of clinical protocols to strategic lobbying for regulatory flexibility and active participation in health technology assessments (HTA). Societal engagement also encompasses individual-level contributions, such as participation in clinical trials and patient preference studies, which increasingly inform regulatory decisions and the evaluation of therapeutic value. In some cases, advocacy groups have not only accelerated access to treatment but also redefined what counts as meaningful clinical benefit, thereby influencing the evidentiary standards of regulatory agencies (7, 8). These developments have been especially visible in the domain of neuromuscular disorders, where organized patient movements, such as those in Spinal Muscular Atrophy (SMA) and Duchenne Muscular Dystrophy (DMD), have built longstanding alliances with researchers, clinicians and public authorities.

For the purposes of this review, we define “societal participation” as the active involvement of non-institutional actors (patients, caregivers, advocacy groups, and civil society organizations) in processes across the orphan drug lifecycle, from research prioritization to regulatory assessment and access negotiations. To structure this analysis, we distinguish four broad categories of actors: (i) patients and caregivers providing direct experiential expertise, (ii) patient advocacy groups (PAGs) offering collective representation, (iii) civil society organizations or NGOs addressing broader societal interests, and (iv) hybrid policy and HTA stakeholders integrating patient voices within evaluative frameworks. This operational definition ensures conceptual clarity and facilitates the mapping of these categories onto the functional roles identified later in the review.

However, while the literature includes various case reports and conceptual reflections on the value of societal engagement, a systematic synthesis of its modalities, outcomes, and limitations remains lacking. Most existing analyses focus on individual conditions or national contexts, and there is limited comparative understanding of the functions that societal actors perform across different stages of OD development. Furthermore, critical dimensions such as legitimacy, representativeness, governance boundaries, social responsibility and long-term sustainability are seldom examined systematically. This review seeks to address this gap by offering a structured synthesis of the peer-reviewed literature on societal participation in OD development, with a particular focus on neuromuscular and neurodegenerative disorders. It pursues three objectives: first, to identify and categorize the diverse forms of engagement undertaken by societal actors; second, to assess the documented impacts of these engagements on the clinical, regulatory, and ethical dimensions of drug development; and third, to examine the risks, gaps, and unresolved tensions that may affect the legitimacy, equity, or sustainability of these participatory models. In doing so, the review also highlights emerging opportunities for expanding and strengthening societal contributions to therapeutic innovation.

## 2 Methodology

### 2.1 Research strategy

This systematic review was conducted in accordance with the PRISMA 2020 guidelines. The primary aim was to explore the forms and impact of societal participation in the development of ODs, with a particular focus on neuromuscular disorders such as SMA.

Two electronic databases, PubMed and Scopus, were searched systematically to identify relevant literature published between January 2000 and March 2025. The final search was performed on March 27, 2025. The same following Boolean search strategy was applied in both PubMed and Scopus using free-text keywords. No controlled vocabulary or field-specific were used. The search string was entered directly in each database’s general search interface. The full search queries as implemented are provided in Appendix A.

No language restrictions were applied during the initial search. Only articles published in English or French were retained for full-text screening. Additional records were identified through manual screening of references and gray literature sources, particularly via Google Scholar and Orphanet. In addition, policy and HTA repositories (EU Publications Office, WHO IRIS, EMA, NICE) were systematically searched to capture non-indexed documents and policy-oriented literature.

To ensure conceptual clarity, we applied an operational definition of societal participation, understood as the active involvement of non-institutional actors (patients, caregivers, advocacy groups, and civil society organizations) across the orphan drug lifecycle. Studies were included only if they explicitly addressed such participatory roles in research, clinical development, regulatory dialog, or access negotiations.

### 2.2 Eligibility criteria

The inclusion and exclusion criteria were defined *a priori* to ensure that only studies directly relevant to the research objectives were retained. Eligible records had to document concrete instances of societal participation in the development of orphan drugs, such as patient-driven funding initiatives, advocacy-led research design, or structured involvement in regulatory and HTA processes. This review focused on rare diseases broadly, while acknowledging that neuromuscular disorders are prominently represented in the evidence base. This reflects the maturity of patient involvement in these conditions rather than a deliberate restriction to a single disease group. No formal geographical limits were applied at the search stage; however, the majority of included studies originated from North America and Western Europe, which we recognize as a source of potential bias and explicitly discuss as a limitation. Only empirical studies, case reports, and program evaluations published in peer-reviewed journals between January 2000 and March 2025 were eligible. Studies were considered irrespective of methodology (qualitative, quantitative, or mixed methods), provided they included a participatory component. Opinion pieces, purely theoretical articles, and technical/molecular research without stakeholder engagement were excluded.

Policy and HTA reports (e.g., EMA, NICE, EU publications) were not included in the formal systematic review due to their heterogeneity and non-peer-reviewed nature. Instead, such documents were screened separately and integrated into the Discussion to contextualize the policy landscape. Table 1 provides a detailed overview of the inclusion and exclusion criteria.

**Table 1:** Inclusion and exclusion criteria used for study selection.

Inclusion criteria	Exclusion criteria
Peer-reviewed empirical studies, case reports, or program evaluations	Studies unrelated to rare diseases or drug development
Articles explicitly addressing societal involvement (e.g., advocacy, lobbying, patient-driven funding, co-design, regulatory or HTA engagement) in the context of orphan drug development	Purely technical or molecular research without a participatory dimension
Studies related to rare diseases (with particular attention to neuromuscular disorders, though not restricted to them)	Opinion pieces, narrative commentaries, or expert perspectives without empirical or documented case content
Publications between 2000 and 2025, in English or French	Non-peer-reviewed reports, unless used solely for policy context in the Discussion

### 2.3 Study selection process

All retrieved records were exported into Excel for screening. After removal of duplicates, titles and abstracts were independently reviewed by two researchers (SA and HS). Potentially eligible articles then underwent full-text screening based on predefined inclusion and exclusion criteria. Disagreements were resolved through discussion and consensus.

The selection process is summarized in the PRISMA 2020 flow diagram (Appendix A). A total of 21 studies were included for full qualitative assessment and synthesis. These articles reflect a broad spectrum of methodological designs, ranging from cross-sectional surveys and observational studies to structured case reports, qualitative interviews, and policy analyses.

Each study was appraised using an appropriate validated tool: AXIS for cross-sectional designs, CASP for qualitative investigations, ROBINS-I for observational analyses, and the JBI checklist for case reports and expert-informed policy reports. Studies lacking empirical methods, such as policy commentaries and expert perspectives, were not subject to formal critical appraisal but were retained for their conceptual relevance.

In total, 16 empirical studies were critically appraised, with quality ratings ranging from moderate to high depending on methodological transparency, sampling, and triangulation. Six non-empirical contributions (commentaries, perspectives, or policy syntheses) were not formally appraised but were analyzed separately for their conceptual and illustrative value. Appendix B provides a detailed summary of the appraisal process and quality ratings assigned to each included study.

### 2.4 Data extraction and synthesis

A standardized data extraction table (cf. Table 2) was created to capture key information from each included article. Data were synthesized narratively to reflect the diversity of participatory models and their influence on regulatory and clinical outcomes. Particular emphasis was placed on the evolving roles of patient advocacy organizations, families, and non-profit funders.

**Table 2:** Data extraction summary across 22 studies.

Reference	Country	Study type	Disease area	Actors involved	Role of societal participation	Type of societal participation
Bertini et al. (9)	France, EU	RCT (Phase 2)	SMA Type 2/3	AFM-Téléthon [PAG], Trophos SA [Industry]	Funding and design support of trial for Olesoxime	1, 2
Gusset et al. (29)	EU	Patient survey	SMA	SMA Europe [PAG]	Assessed patients’ expectations toward therapy	3, 5
Patterson (20)	USA	Program implementation	SMA	Cure SMA [PAG]	Created trial-readiness tools co-developed with families	2
Peay et al. (18)	USA	Qualitative study	SMA / DMD	Parents [Patients/Caregivers], Advocacy groups [PAG]	Explored trial participation facilitators/barriers	3, 5
Tizzano et al. (16)	Global	Program report	SMA	Researchers [Academics], SMA orgs [PAG]	Education and engagement to improve trial quality	2
Gaillard et al. (10)	France	Case study	Cystinosis	Necker [Academics], Orphan Europe [Industry], Families [Patients/Caregivers]	Collaboration across academics, pharma, and patient orgs	3
Bird et al. (19)	UK	Commentary / Viewpoint	Neuroblastoma	Patient advocates [PAG]	Advocacy shaping priorities and funding	3
Patterson (20)	Global	Review / Commentary	Rare diseases	PAGs [PAG]	Emphasized strategic role of patient advocacy	3, 4
Nguyen et al. (13)	Australia	Narrative review	Pediatric neurology	RDPO leaders [PAG]	Showed how RDPOs shape early-stage innovation	1, 3
Frost et al. (24)	UK	Qualitative synthesis	ODs	Mixed patient populations [Patients/Caregivers]	Captured diversity in patient engagement	3, 4, 5
Huml et al. (23)	USA	Case report	Muscular Dystrophies	MD-specific orgs [PAG]	Demonstrated long-term impact of PAGs	3
Aartsma-Rus et al. (8)	EU / USA	Review / Opinion	Rare diseases	Advocacy groups [PAG], Scientists [Academics]	Argued for early patient involvement in R&D	2, 4
Epps et al. (14)	Global	Policy analysis	Pediatric rare diseases	Regulators [Hybrid/HTA], Families [Patients/Caregivers], NGOs [Civil Society]	Described policy push for patient-centered trials	1, 2, 3
Reichel et al. (12)	Germany	Review	Multiple rare diseases	Patient orgs [PAG], Donors [Civil Society]	Focused on philanthropic drug development	1, 4
Stein et al. (25)	USA	Guideline development	Rare diseases	Advocacy orgs [PAG], Industry [Industry]	Developed ethical framework for PAG–pharma interactions	2, 3
Daban et al. (27)	France	Case example	Rare diseases	LMC France [PAG]	Involvement of patients in therapeutic information sharing	3, 4
Wicks et al. (21)	USA	Observational / Self-initiated trial	ALS	ALS patients (via PatientsLikeMe) [Patients/Caregivers]	Patient-led trial using online community platform	3, 4
Mavris and Le Cam (22)	EU	Survey of patient groups	Rare neuromuscular diseases	EURORDIS members [PAG], AFM-Téléthon [PAG]	Funding, trial recruitment, co-design of research	3, 4
Furlong et al. (11)	USA / Global	Case study	DMD	PPMD [PAG]	Patient-led development of FDA guidance	1, 3
Furlong et al. (17)	USA / UK	Program co-design	DMD, DM1	Dyne Therapeutics [Industry], Patient advisory boards [PAG/Patients]	Co-creation of trials and logistics with families	2, 4, 5
Jimenez-Moreno et al. (28)	EU	Qualitative study	Rare neuromuscular diseases	Patients [Patients/Caregivers], Caregivers [Patients/Caregivers], Clinicians [Academics]	Patient preference elicitation	5
Pickaert (26)	EU	Review / qualitative desk research	Cross-disease (HTA of medicines)	Patient organisations [PAG], HTA bodies (NICE, SMC, HAS, IQWiG/G-BA, AIFA, AEMPS, CADTH/CDA-AMC) [Hybrid/HTA]	Advocacy for JCA framework and transparency; Guidance/resources, plain-language outputs; Patient input to outcomes/comparator selection	3, 4, 5

22 studies: Country, Study Type, Disease Area, Actors Involved, Role of Societal Participation, and Mapping to Five Participation Categories—(1) Patient-Driven Funding, (2) Co-Design of Clinical Trials and Readiness Tools, (3) Advocacy and Lobbying for Regulatory Approval and Market Access, (4) Informational and Educational Contributions, and (5) Patient-Centered Decision-Making via Preferences and Lived Experience.

## 3 Results

### 3.1 Forms of societal participation identified across studies

This systematic review identified a wide array of societal involvement mechanisms in the development of ODs, cutting across diverse disease areas, geographical settings, and stages of the drug development lifecycle. Analysis of the 22 selected studies (cf. Table 2) revealed five recurrent categories of societal participation: *(1) patient-driven funding initiatives supporting early-stage research, (2) co-design of clinical trials and readiness tools aimed at improving recruitment and relevance, (3) advocacy and lobbying efforts to influence regulatory approval and market access, (4) informational and educational contributions to enhance stakeholder understanding and engagement, and (5) active shaping of patient-centered trial decision-making, particularly through preference elicitation and the integration of lived experiences*. Several studies combined multiple forms of societal participation simultaneously, illustrating that patient and community actors often play overlapping roles, such as funders, trial collaborators, policy advocates, within the same initiative. This reflects the complex and evolving nature of their involvement across the OD development process.

#### 3.1.1 Patient-driven funding and philanthropic investment

Several studies highlighted the pivotal role of patient organizations in directly financing early-stage research and clinical trials for rare diseases. A well-documented example is the development of Olesoxime for spinal muscular atrophy, where AFM-Téléthon, a French advocacy group, partnered with Trophos SA to support early-phase trials, making proof-of-concept possible (9). Similar mechanisms were observed in other contexts: in the United States, the Cystic Fibrosis Foundation invested through a venture philanthropy model that proved decisive for the development of ivacaftor, while in Europe the AKU Society coordinated the pan-European DevelopAKUre consortium, enabling the repurposing of nitisinone for alkaptonuria (7, 10). These cases illustrate how philanthropic and patient-driven financing can complement or substitute for conventional pharmaceutical investment, particularly in rare and ultra-rare conditions where commercial incentives are weak.

Beyond such examples, other studies have examined philanthropic drug development models more broadly, describing them as complementary or alternative pathways to conventional pharmaceutical investment. For instance, Furlong et al. described the involvement of rare disease foundations in multistakeholder research consortia, in which patient groups acted as co-funders and strategic facilitators throughout the development process (11). Reichel et al. reported similar cases in which patient organizations structured funding partnerships with public and private actors to advance candidate therapies, particularly in domains with low commercial attractiveness (12). Nguyen et al. also documented the use of biomedical venture philanthropy by patient groups to support translational research, fund early-stage clinical trials, and coordinate with academic investigators and regulatory stakeholders (13). In a broader policy analysis, Epps et al. highlighted the role of patient-led organizations in shaping funding priorities and directly supporting trial infrastructure, particularly in pediatric rare disease contexts (14).

Across these studies, patient-driven financial contributions were identified at various stages of drug development, most frequently at the preclinical and early clinical phases, and were often associated with rare or ultra-rare disease indications where conventional investment was limited (11–14).

#### 3.1.2 Co-design of trials and readiness infrastructure

Another frequently documented mode of societal engagement concerns the co-construction of trial protocols, readiness tools, and implementation infrastructure. In the case of SMA, Cure SMA, a US-based advocacy organization, developed a Clinical Trial Readiness Program designed to standardize assessments and enhance site preparedness. The program included a virtual readiness evaluation, a dedicated toolkit for research coordinators and physical therapists, and tailored training activities focused on patient-centered conduct and site compliance (15).

At the international level, SMA Europe initiated a face-to-face educational program targeting clinicians and physiotherapists in countries with limited trial experience. The initiative emphasized protocol interpretation, ethical readiness, and communication with families. It combined theoretical modules with role-play simulations and was developed in collaboration with patient representatives and sponsors to prepare emerging sites for multicenter trials (16).

Beyond neuromuscular disorders, co-design initiatives have also been reported in the context of DMD and myotonic dystrophy. Furlong et al. described how community advisory boards contributed to the refinement of trial inclusion and visit criteria, the design of travel and rest arrangements, and the development of age-appropriate informed consent materials for pediatric and adolescent participants (17).

Several studies also referenced existing infrastructures and transnational platforms that support patient co-design at scale. Among these, the European Patients’ Academy on Therapeutic Innovation (EUPATI) was mentioned as a central initiative promoting training and structured involvement of patients in industry-led protocol development and regulatory dialog (8).

#### 3.1.3 Advocacy and political mobilization

Multiple studies emphasized the strategic role of advocacy in mobilizing political will, shaping regulatory agendas, and catalyzing the development of ODs. Peay et al. conducted a qualitative study showing how family-led initiatives in SMA and DMD contributed to overcoming barriers such as institutional mistrust and insufficient trial literacy, ultimately facilitating trial enrollment (18). Similarly, Bird et al. documented the capacity of neuroblastoma advocates to redirect research priorities and foster partnerships between families and researchers in response to perceived neglect by commercial sponsors (19).

Beyond disease-specific efforts, broader trends were highlighted across diverse rare conditions. Patterson et al. reported that many PAGs now engage proactively in translational research, contributing to natural history studies, policy advocacy, and biobanking infrastructures (20). Epps et al. and Nguyen et al. identified lobbying efforts related to regulatory equity, reimbursement reform, and ethical trial access as core functions of PAGs in the pediatric rare disease ecosystem (13, 14). These actions often intersect with structured networks such as EUPATI, which plays a central role in educating and equipping patients for informed regulatory engagement.

Wicks et al. offered a striking example of patient-led mobilization in ALS, where individuals initiated observational studies via online platforms (*PatientsLikeMe*) prior to any formal regulatory assessment, thereby generating real-world data that anticipated the outcomes of subsequent randomized trials (21). Similarly, Mavris and Le Cam documented how EURORDIS-facilitated coalitions leveraged collective advocacy to influence early-stage research design and policy formation (22). In the context of cystinosis, Gaillard et al. described a multistakeholder initiative in which families, clinicians, and pharmaceutical partners jointly contributed to redefining care and trial structures (10). Huml et al. (reported on long-term interactions between patient organizations and regulators in the field of muscular dystrophies, with advocacy actors participating in guidance development and policy consultation processes (23).

Furlong et al. detailed how patient organizations contributed directly to shaping FDA guidance documents, while Frost et al. and Stein et al. underscored the rising legitimacy of advocacy voices in both US and EU regulatory settings (11, 24, 25). This shift from passive beneficiaries to active co-architects of the innovation pathway was consistently reported across the included literature. In Europe, these advocacy efforts have also been progressively institutionalized. Since the early 2000s, patient representatives have held full membership and voting rights in the EMA’s Committee for Orphan Medicinal Products (COMP), and have contributed systematically to scientific advice through the Patients’ and Consumers’ Working Party, thereby embedding patient expertise within formal regulatory deliberations (22).

In a complementary policy-focused study, Pickaert examined how PAGs have influenced HTA processes across multiple European jurisdictions. The analysis showed that advocacy actors increasingly contribute to the legitimacy and procedural transparency of joint clinical assessments (JCAs), particularly by framing outcome relevance and comparator justification as advocacy priorities (26).

#### 3.1.4 Participation in information dissemination and public education

Beyond funding and trial design, societal actors also contributed through the production and dissemination of therapeutic knowledge, often filling gaps left by industry, academia, public agencies, or non-governmental organizations (NGOs). In several studies, patient organizations were described as uniquely positioned to translate complex medical and regulatory content into accessible language, drawing on their lived expertise and proximity to affected families. A case study in France involving LMC France, a chronic myeloid leukemia advocacy group, reported how patient-parent organizations contributed to the dissemination of drug information and therapeutic options, thereby enhancing public understanding and supporting informed treatment decisions (27).

These informational interventions were particularly relevant in contexts where official documentation was perceived as overly technical or inaccessible. Frost et al., in a qualitative synthesis, noted that patients frequently expressed the need for lay summaries, trial result explanations, and transparent communication channels to better understand the meaning and impact of their participation (24). This finding aligns with the review by Pickaert, which documented how patient organizations systematically produce plain-language summaries, methodological toolkits, and feedback reports to enhance accessibility of HTA outputs across diverse audiences (26).

Several studies described the use of co-created materials (newsletters, infographics, or educational guides) as a strategy to both increase health literacy and legitimize patients’ perspectives in the research process.

Platforms such as *PatientsLikeMe* enabled direct, patient-led dissemination of observational findings and treatment experiences, contributing to a growing ecosystem of decentralized knowledge-sharing (21). Other studies highlighted the role of patient groups in supporting the interpretation of genomic data, constructing accessible consent documentation, or designing orientation materials to accompany trial participation (17, 20).

#### 3.1.5 Active shaping of patient-centered trial decision-making

Several studies underscored the role of societal actors in shaping clinical trial decision-making by articulating patient preferences, expectations, and tolerance for uncertainty or risk. These contributions were especially salient in early-stage or understudied conditions, where conventional clinical endpoints may not align with patients’ lived priorities.

Jimenez-Moreno et al. conducted a qualitative study involving patients with myotonic dystrophy and mitochondrial myopathies to identify the treatment attributes and risks most meaningful to participants (28). The study revealed shared priorities across diseases, like muscle strength, energy, and endurance, as well as differentiated tolerance thresholds for specific adverse events. These insights were used to design a subsequent preference-based experiment tailored to patient-defined outcomes (28).

In a related context, Gusset et al. reported the results of a pan-European survey among individuals with SMA, documenting expectations regarding functional improvement, treatment goals, and the perceived trade-offs between efficacy and burden (29). These data were explicitly collected to inform regulatory, reimbursement, and trial planning efforts by SMA Europe.

At a regulatory interface, Pickaert highlighted how structured patient-experience inputs are increasingly being incorporated into HTA deliberations, particularly in defining relevant outcomes and comparator choices in JCAs (26). This illustrates how preference elicitation is moving beyond research contexts into formal evaluative frameworks.

Furlong et al. provided evidence of patient and caregiver feedback being integrated into the operational design of clinical trials for Duchenne and myotonic dystrophy. Community Advisory Boards were engaged to refine inclusion criteria, structure study visits, and ensure age-appropriate consent materials. In parallel, preference-sensitive elements such as travel burden, recovery time, and anxiety mitigation in pediatric populations were addressed through logistical adaptations (17).

Peay et al. described how family perspectives influenced engagement strategies and retention in pediatric neuromuscular trials, emphasizing the need for context-sensitive trial planning and transparent communication around benefit–risk expectations (18).

Frost et al. highlighted how participant testimonies from rare disease trials revealed gaps in responsiveness to patient-expressed endpoints, particularly for those not enrolled in trials or excluded from early access (24). The study also noted the relative absence of frameworks for incorporating patient-defined success criteria into endpoint validation (24).

Collectively, these studies demonstrated that eliciting and integrating patient preferences has become an operational feature of trial design in rare diseases, particularly in areas where clinical uncertainty or small sample sizes preclude rigid standardization.

### 3.2 Reported outcomes of societal participation in orphan drug development

Over the past decades, societal participation shifted from a passive to an active role in the drug development process. The analysis of the selected studies reveals that societal participation has produced diverse and multifaceted impacts, extending far beyond participating in clinical trials to designing innovative trial protocols, and sharing regulatory, ethical, and dissemination frameworks. These effects can be categorized into six key outcome areas: (1) acceleration of development timelines, (2) facilitation of recruitment and participant retention, (3) increased quality and relevance of endpoints, (4) leverage in Health Policy and Economic Evaluation, (5) public-facing dissemination and increased health literacy, and (6) long-term systemic change.

#### 3.2.1 Acceleration of drug development and trial initiation

Several articles reported that societal engagement contributed to significantly faster initiation of clinical trials, particularly in contexts where traditional pharmaceutical sponsors were hesitant to invest. For instance, the development of Olesoxime for SMA, funded by AFM-Téléthon, reached phase 2 trials much earlier than expected due to pre-existing collaborations between families, researchers, and biotech firms (9). Similar mechanisms were described in the field of muscular dystrophies, where sustained advocacy pressure prompted regulators to expedite review timelines and grant early access to investigational therapies (23).

This acceleration is further supported by the emergence of trial readiness platforms, such as those designed by Cure SMA in the US, which enabled rapid patient enrollment and minimized administrative bottlenecks (15). Mavris and Le Cam also reported how early engagement of EURORDIS members in protocol design and recruitment strategy enabled clinical studies for neuromuscular diseases to advance more quickly through institutional review phases (22).

In a digital context, Wicks et al. described how patients with ALS used the *PatientsLikeMe* platform to self-organize and complete an online observational study ahead of any formal randomized trial (21). Their results, publicly disseminated in advance, matched subsequent RCT outcomes and demonstrated how decentralized patient mobilization can pre-empt traditional timelines.

Finally, Furlong et al. noted that the co-development of disease-specific guidance documents between the FDA and patient groups led to more predictable and time-efficient regulatory interactions in the DMD space (11).

#### 3.2.2 Recruitment facilitation and participant retention

Several studies reported that the involvement of patient organizations and family networks helped facilitate the recruitment process in clinical trials for rare diseases. In one qualitative study, parents of children with neuromuscular disorders described how trust in patient organizations served as a contextual anchor that made clinical participation more comprehensible and less intimidating (18). These findings were echoed in multi-country initiatives where advocacy groups provided tailored communication strategies and peer-based reassurance, particularly in pediatric contexts where consent and comprehension dynamics are complex (16).

Furlong et al. noted that the co-design of participant-facing materials (including travel guidance, rest planning, and consent forms adapted to developmental stages) helped reduce logistical friction and uncertainty for families enrolled in trials for Duchenne and myotonic dystrophy (17). These structural contributions may not alter a patient’s intrinsic willingness to participate but were reported to support clearer decision-making and higher follow-through.

These adaptations were also associated with improved retention rates, as seen in a European SMA survey where dropouts were often linked to unmet expectations or unclear communication around burden and benefit (29). Similarly, Jimenez-Moreno et al. described how alignment between trial demands and patients’ preferred outcomes was perceived to sustain motivation throughout the study period (28).

Across these examples, societal actors did not necessarily convince patients to participate, but played a key role in enabling, clarifying, and supporting their engagement under conditions of uncertainty.

#### 3.2.3 Enhancement of trial design quality and endpoint relevance

Societal input was also shown to improve the clinical relevance and acceptability of trial endpoints. In many cases, families co-developed outcome measures that reflected their lived experience with the disease, the so-called PROMs and PREMs (Patient-Reported Outcome Measures and Patient-Reported Experience Measures), complementing purely biomedical indicators. This was particularly important in rare pediatric settings, where traditional efficacy metrics may overlook meaningful changes in quality of life or caregiver burden (24).

In pediatric neurology, patient organizations contributed to identifying age-appropriate, functionally significant outcomes that had previously been neglected by sponsors (13). These contributions were acknowledged by sponsors and regulators, suggesting a maturing role for advocacy in shaping what counts as valid evidence.

Jimenez-Moreno et al. further demonstrated how qualitative research involving patients with myotonic dystrophy and mitochondrial myopathies led to the identification of preferred treatment attributes, including muscle strength, energy levels, and fatigue mitigation (28). These results informed the construction of subsequent quantitative preference studies aimed at aligning endpoints with patient-defined priorities.

Pickaert reinforced this perspective by showing how patient submissions to HTA bodies and joint clinical assessments contributed to refining which outcomes and comparators were considered relevant for evaluation, thereby aligning methodological standards more closely with patient-experienced priorities (26).

Furlong et al. reported on the involvement of the Duchenne community in developing a disease-specific guidance document with the US FDA, which included discussions on endpoints that were both clinically and experientially relevant to families. The resulting document contributed to the formal recognition of functional measures such as time-to-stand and six-minute walk test, which had not been prioritized in earlier regulatory standards.

#### 3.2.4 Leverage in health policy and economic evaluation

A less visible, yet critical, outcome of societal participation lies in its growing role within health policy processes and early value assessment frameworks. As noted in the SMA Europe survey, patients’ expectations and therapeutic goals were used by national HTA bodies to inform access negotiations for high-cost therapies (29). In some cases, advocacy-generated evidence has served as a source of gray literature cited in reimbursement dossiers and public consultations (14).

In the European context, Mavris and Le Cam described how EURORDIS patient representatives contributed to regulatory and HTA dialogs via their participation in IRDiRC and EMA scientific committees (22). These activities included the formulation of methodological positions on patient relevance and early treatment value, upstream of formal cost-effectiveness evaluations.

Furlong et al. reported that the Duchenne community collaborated with the US FDA to co-author guidance documents that later influenced how economic and clinical endpoints were framed in US payer discussions (11). In parallel, Jimenez-Moreno et al. outlined how qualitative preference studies established by patients with neuromuscular diseases informed the selection of attributes for future quantitative valuation tools—tools often used in early benefit–risk and pricing models (28).

Wicks et al. demonstrated that community-led observational data collection could precede and inform formal trials, providing regulators and decision-makers with contextualized real-world signals, even in the absence of commercial sponsorship (21).

Complementing these findings, Pickaert documented how patient organizations actively shaped HTA deliberations in Europe, particularly within the emerging JCA framework. Their interventions extended beyond testimonies, influencing procedural transparency and methodological framing around outcome relevance, comparator choice, and equitable access (26).

While these actions may not directly generate economic savings, they contribute to earlier evidence development and better alignment of regulatory and reimbursement priorities with patient-defined value.

#### 3.2.5 Public-facing dissemination and increased health literacy

Information sharing, capacity-building, and dissemination efforts emerged as critical roles for patient organizations. Several studies highlighted the creation of educational resources and information platforms co-developed by patients, aimed at improving understanding of clinical trials and therapeutic options (20, 27). These efforts included newsletters, webinar series, explanatory videos, and consent documents simplified through iterative feedback with affected families.

Wicks et al. described how patients with ALS used the *PatientsLikeMe* platform to share treatment experiences and trial outcomes with other community members, contributing to a model of direct, real-time knowledge circulation (21). The observational data generated through this peer-based mechanism also mirrored the outcomes of subsequent RCTs, underscoring its dual role in patient education and evidence production.

Furlong et al. reported that advocacy groups involved in FDA guidance development for DMD simultaneously produced accessible versions of regulatory documents, including annotated summaries, visual tools, and parent-directed FAQs to improve comprehension and public uptake (11).

Mavris and Le Cam described the use of targeted training programs organized by EURORDIS to build patient capacity to participate in scientific committees, thereby equipping them not only to access complex information but also to act as intermediaries between research teams and their communities (22). In this vein, Pickaert highlighted that patient groups increasingly produce plain-language summaries, methodological guides, and feedback reports on HTA processes (26). By translating technical assessments into accessible outputs, they enhanced community health literacy and fostered more informed participation in subsequent policy debates.

Across these cases, dissemination was not limited to passive access to information, but rather oriented toward enhancing patient and caregiver literacy, enabling them to evaluate trade-offs, ask informed questions, and participate actively in shared decision-making.

#### 3.2.6 System-level changes and ethical standard setting

Finally, some forms of societal engagement appear to have catalyzed structural transformations in the orphan drug development ecosystem. Advocacy coalitions were directly involved in the co-creation of ethical guidelines for interactions between patient organizations and pharmaceutical companies, helping to formalize norms on transparency, governance, and conflict of interest management (25).

Aartsma-Rus et al. further described the institutionalization of patient representation in transnational consortia, leading to the routine inclusion of advocacy input in research design and regulatory dialog (8).

Several studies also documented how these engagements led to durable governance structures. Mavris and Le Cam described how patient representatives gained full voting rights within the EMA’s Committee for Orphan Medicinal Products (COMP), and have maintained leadership positions such as vice-chair over multiple cycles (22). This institutional shift was accompanied by the creation of patient-led research agendas and involvement in strategic planning for the IRDiRC and EUCERD frameworks.

Furlong et al. reported that the co-development of guidance documents with the FDA not only influenced individual protocols but also altered procedural expectations around patient involvement, leading to the incorporation of community review into standard regulatory workflows (11).

Wicks et al. showed that decentralized, patient-driven observational research could precede traditional R&D structures, and indirectly prompt reconsideration of how early-stage data and patient-generated evidence are integrated into regulatory science (21).

More recently, Furlong et al. described how community advisory boards engaged in trial co-design became standing partners in sponsor review processes, laying the foundation for a hybrid governance model that spans protocol, ethics, and dissemination oversight (17).

Together, these studies illustrate how societal participation has contributed to redefining the ethical and procedural frameworks governing orphan drug development, moving from consultative models to sustained institutional roles.

### 3.3 Functional typology of societal participation in orphan drug development

**Figure 1:**
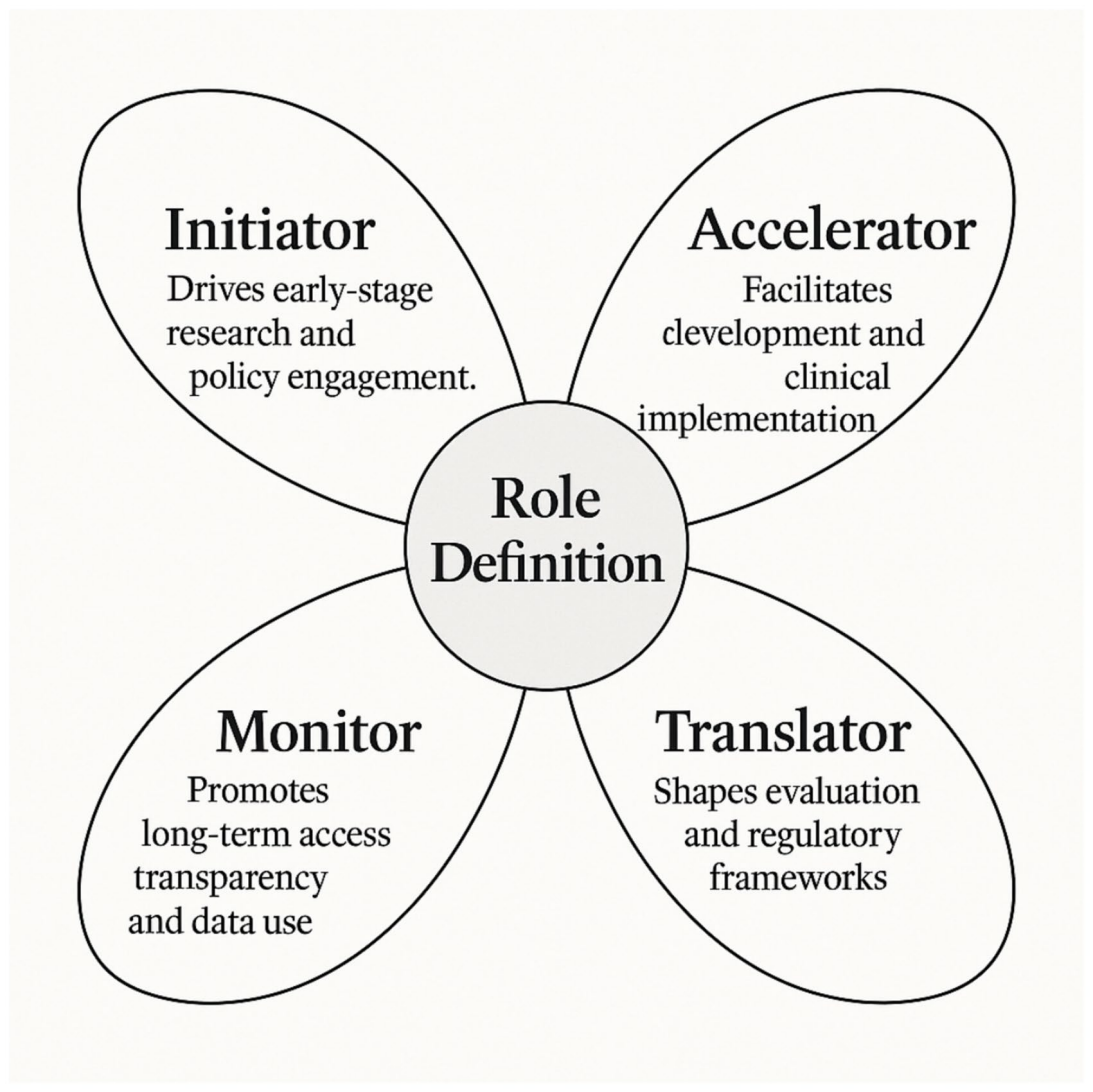
Functional typology of societal participation in orphan drug development. This original conceptual model, developed from the findings of this systematic review, distinguishes four complementary roles “Initiator, Accelerator, Translator, and Monitor” through which societal actors contribute across the OD development pathway.

Beyond listing individual contributions or measured outcomes, the included studies collectively support the idea that societal participation in OD development is not a monolithic phenomenon. Instead, it assumes multiple functions depending on the disease context, the maturity of the research ecosystem, and the degree of institutionalization of patient advocacy. Based on the cross-comparative synthesis of the 22 articles, we propose a four-role functional typology (cf. Figure 1) that categorizes societal participation into four non-exclusive, yet analytically distinct roles: *Initiator*, *Accelerator*, *Translator*, and *Monitor*. A summary of the main outcomes and illustrative examples for each role is provided in Supplementary Table S1.

#### 3.3.1 Initiator: triggering development pathways

In several cases, societal actors play the role of development initiators, launching or resuscitating drug development programs in the absence of sufficient commercial or academic interest. This was most evident in the case of Olesoxime, where the AFM-Téléthon provided early-stage funding and partnered with biotechnology actors to support clinical trials (9). Similarly, patient-driven funding mechanisms, described in philanthropic models, were instrumental in triggering R&D in neglected ultra-rare indications (12).

Such initiation often involves early risk-sharing, coordination of research priorities, and mobilization of dispersed expertise, a role that would traditionally fall under the scope of governmental or commercial funders.

#### 3.3.2 Accelerator: reducing bottlenecks in clinical trials

Other organizations assume the role of accelerators, addressing the operational and administrative challenges that typically delay OD trials. This included the development of trial readiness platforms, logistical support for participant identification, and the creation of pre-consent materials tailored to families (15, 16).

By improving trial infrastructure and increasing patient literacy, these actors helped reduce recruitment delays and enhanced retention, ultimately shortening the timeline from concept to data. This role is particularly critical in pediatric diseases, where timing is often linked to irreversible disease progression.

#### 3.3.3 Translator: aligning research with patient-relevant endpoints

Societal participation also serve a translational function, whereby lived experience and community preferences informed the design, measurement, and interpretation of trial outcomes. This translation from patient experience to scientific evidence not only enhances the relevance and acceptability of clinical endpoints but also strengthens the ethical robustness of research protocols. Importantly, translation extends beyond trial settings: advocacy organizations increasingly contribute to the interpretation and communication of HTA outcomes, producing plain-language summaries and providing patient-experience inputs that frame which endpoints and comparators are deemed meaningful in evaluation processes (13, 24, 26). In this sense, advocacy actors act as epistemic bridges not only between patients and researchers, but also between technical appraisal bodies and the wider community.

#### 3.3.4 Monitor: promoting ethical governance and accountability

Finally, societal actors assume monitoring functions, engaging in critical reflection on the ethical and political dimensions of drug development. Beyond drafting ethical guidelines or lobbying for equitable access, their involvement increasingly extends to the governance of health technology assessment, where advocacy groups call for transparency, methodological inclusiveness, and systematic consideration of patient-reported priorities (25, 26, 29).

By participating in governance mechanisms, watchdog coalitions, or HTA discussions, these actors have evolved from support groups to accountability stakeholders, shaping not only drug availability but also the terms of its social legitimacy.

### 3.4 Risks, gaps, and unresolved tensions in societal participation

While the reviewed studies document a wide range of benefits associated with societal engagement in OD development, several recurring limitations and unresolved tensions were identified. These issues concern the scope, legitimacy, equity, and sustainability of such participation, and warrant critical reflection before societal models are further scaled or institutionalized.

#### 3.4.1 Representation and geographic inequities

A fundamental concern relates to who participates and whose voices are amplified. Many of the studies reviewed highlight the pivotal role of well-organized PAGs; however, such groups may not always represent the diversity of patient experiences, particularly in fragmented or newly diagnosed communities (18, 24). Selection biases, organizational hierarchies, and language or cultural barriers can all restrict inclusivity, risking a reproduction of inequalities in who shapes drug development priorities.

The studies also suggest that societal engagement is unevenly distributed across disease areas and geographic regions. Conditions with strong, long-standing advocacy networks (e.g., SMA, DMD) have attracted considerable participatory investment, whereas ultra-rare, low-profile disorders remain largely underrepresented, or are only indirectly represented through umbrella organizations such as RadiOrg (12). Similarly, most documented cases originate from North America, Europe, or Australia, with limited evidence from low- and middle-income countries. Some efforts, such as transnational patient representation at EMA or digital platforms enabling broader self-mobilization, have attempted to reduce these gaps, but their reach remains uneven (21, 22).

#### 3.4.2 Risks of tokenism and instrumentalization

Another recurrent theme identified across the reviewed literature concerns the risk of tokenistic or instrumental forms of patient engagement. While several studies acknowledged that societal input can improve ethical and methodological standards (25), others emphasized the possibility that patient participation may be co-opted by commercial actors to serve pre-existing strategic goals (20). This form of tokenism typically occurs when engagement is introduced only at late stages of development or in highly curated formats, without offering patients meaningful influence over core decisions.

Such practices include inviting advocacy representatives into advisory boards with limited mandate, soliciting personal narratives primarily for communication or pricing justifications, or using participation metrics as a proxy for legitimacy in regulatory submissions. These dynamics risk undermining the credibility of participatory processes and may contribute to patient fatigue or distrust over time.

#### 3.4.3 Blurred roles and expectations

Some forms of participation challenge traditional boundaries between stakeholder roles, leading to ambiguity around responsibilities, authority, and accountability. When patient organizations contribute to endpoint selection, trial logistics, and funding; as observed in the development of Olesoxime or readiness infrastructure; they may also face pressure to assume quasi-regulatory or scientific roles for which they are not institutionally equipped (9, 15).

Other studies reported similar overlaps. In the context of FDA guidance development, patient advocates were involved in drafting content and coordinating input from diverse community actors (11). Advisory boards involved in trial co-design were also asked to comment on protocol feasibility, risk management, and communication plans (17). In regulatory settings, patient representatives have participated in formal scientific committees, contributing to discussions that require alignment with procedural norms and evidence standards (22).

Although these observations emerge clearly in the context of neuromuscular and rare diseases, they reflect broader tensions extensively discussed in the wider literature on patient engagement. Initiatives such as EUPATI have emphasized the need for dedicated training and support to avoid role overload and to maintain the legitimacy of experiential knowledge in expert arenas. This suggests that clearer role delineation and formal capacity-building frameworks are required to sustain meaningful participation without disproportionate burden.

#### 3.4.4 Evidence gaps and longitudinal blind spots

Despite the growing number of descriptive and conceptual studies, only a limited subset of the included literature offers longitudinal data or robust outcome evaluations that would enable assessment of long-term impact. Reported effects such as accelerated development or improved recruitment are frequently stated, but often inferred rather than supported by systematically collected data (13, 29). Furthermore, potentially unintended consequences, such as over-medicalization, inequitable access, or misalignment with broader public health priorities, remain largely unexplored.

Studies have begun to address these limitations by focusing on patient preference elicitation as a tool to align development and evaluation processes with patient-defined priorities (28). However, the integration of such data into regulatory or economic decision-making frameworks remains inconsistent across disease areas.

In this context, broader initiatives such as IMI PREFER, a public-private partnership launched under the Innovative Medicines Initiative and developed in close collaboration with the EMA, have sought to establish methodological guidance for the design, conduct, and application of patient preference studies throughout the product lifecycle. The recommendations produced provide a structured foundation to ensure preference data are collected in a rigorous and decision-relevant manner (30). Yet, the long-term impact of such frameworks on regulatory practices, reimbursement decisions, or equitable treatment access still requires further empirical validation.

## 4 Discussion

### 4.1 Societal shifts in innovation

The findings of this review confirm that societal actors have evolved from peripheral stakeholders to central agents in the orphan drug development landscape. Across the studies reviewed, their roles extended far beyond passive consultation, encompassing funding initiation, methodological co-construction, and ethical governance. These shifts reflect a broader reconceptualization of pharmaceutical innovation as a distributed process, no longer confined to industrial pipelines or academic silos but co-produced by lay expertise, political mobilization, and experiential knowledge (24).

This transformation is particularly salient in neuromuscular disorders, where advocacy organizations have acted as de facto sponsors or coordinators of drug development programs, such as in the case of Olesoxime for SMA (9). Rather than functioning as adjuncts to industry-led trials, these actors have shaped priorities, influenced endpoints, and in some cases redefined what constitutes valid evidence or acceptable risk–benefit thresholds (22, 25).

Comparable dynamics are observable across other disease areas. In cystic fibrosis, the Cystic Fibrosis Foundation’s venture philanthropy was decisive in enabling ivacaftor (7). In alkaptonuria, the AKU Society coordinated the DevelopAKUre consortium that brought nitisinone to regulatory approval (10). In ALS, the PatientsLikeMe platform demonstrated the potential of patient-driven real-world data to anticipate and even inform formal trial outcomes (21). Together, these cases exemplify how patient and advocacy groups have not only influenced trial design and evidentiary standards, but have actively reconfigured the governance of innovation across diverse therapeutic areas and regulatory contexts.

### 4.2 Clarifying roles and boundaries

The typology emerging from this review, “Initiator, Accelerator, Translator, and Monitor,” offers an analytical framework to make sense of the multiplicity of societal engagements in drug development. While these roles are often overlapping in practice, distinguishing them conceptually allows for a clearer articulation of responsibilities, boundaries, and required capacities.

For example, “initiator” roles imply financial risk-taking and agenda-setting functions that resemble those of venture funders or public R&D agencies. “Translators,” by contrast, operate as epistemic brokers, ensuring that scientific protocols reflect lived realities and patient-relevant outcomes. This division of functions also has implications for governance: not all patient groups are equipped or should be expected to fulfill all roles simultaneously. Tailored support, role clarification, and equitable collaboration frameworks are therefore needed to avoid overburdening advocacy actors or blurring lines of accountability (18, 25).

These concerns were illustrated in recent co-design programs where advisory boards contributed simultaneously to trial logistics, informed consent form adaptation, communication planning, and participant retention strategies; functions traditionally spread across sponsors, CROs, and clinicians (17). Similarly, in institutional settings such as the EMA’s Committee for Orphan Medicinal Products, patient representatives were called upon to contribute to scientific deliberation while also acting as community spokespersons, raising questions about the structural support needed to sustain such hybrid roles (22).

The boundaries between these roles are increasingly tested in institutional contexts. In HTA deliberations, for example, advocacy groups are simultaneously asked to act as translators of technical evidence and as monitors of accountability, a dual function that risks overextension without adequate resources or training (26).

In addition to functional diversity, our review indicates a clear temporal trajectory in the ways societal actors have contributed over the last decade. Early contributions (2011–2015) were dominated by Initiator functions, including patient-driven funding models, the creation of research cohorts, and the co-development of regulator-facing guidance documents. The mid period (2018–2020) reflects the consolidation of Accelerator and Translator roles, with a focus on trial readiness infrastructures, collaborative protocol design, and the emergence of patient preference studies. From 2021 onward, we observe the institutionalization of participation, with stronger Translator and Monitor functions anchored in methodological guidance, HTA interfaces, and formal participation in committees and advisory structures. Together, these trends suggest a maturation from *ad hoc* agenda-setting toward more structured and governance-linked participation.

### 4.3 Implications for regulation

The growing involvement of societal actors in drug development raises important questions for regulatory science and HTA. While their contributions are increasingly recognized in conceptual debates, they remain only partially institutionalized in formal decision-making processes. As shown in this review, advocacy organizations and patient groups have influenced trial design, endpoint selection, and early access pathways but these interventions are often ad hoc, geographically uneven, and poorly integrated into regulatory frameworks.

Recent policy and HTA repositories complement the peer-reviewed evidence by offering concrete frameworks for operationalizing societal participation in regulatory contexts. For regulatory agencies and HTA bodies, the EU Health Technology Assessment Regulation [EU HTAR; Regulation (EU) 2021/2282, effective 2025] highlights the need to establish predictable procedures for patient involvement at both the scoping and appraisal stages.

These developments make the upcoming implementation of EU HTAR especially timely. From 2025 onward, Joint Clinical Assessments (JCAs) across Member States will adopt the PICOS format (Population, Intervention, Comparator, Outcome, Study design) as the common evaluative structure. This framework explicitly opens new entry points for societal engagement, particularly in defining outcomes that reflect patient priorities and in justifying comparators relevant to lived experience.

Initiatives led by the EUnetHTA21 consortium, tasked with piloting methodologies and templates for EU HTAR, have already identified “Outcome relevance” and “Comparator justification” as domains where patient input is necessary, yet underutilized, especially in rare disease contexts where standard clinical endpoints may fail to capture meaningful benefit. Interim methodological guidelines (EUnetHTA21, 2022–2024) encourage early inclusion of patient-defined outcomes during the scoping phase of JCAs. Similarly, the EMA Patients’ and Consumers Working Party (PCWP) has advocated for more structured and upstream involvement of societal actors in regulatory HTA interfaces. This trend is consistent with recent analyses showing that patient organizations are not only providing preference data but also reframing how HTA bodies interpret outcome relevance and comparator justification, particularly in rare disease settings (26).

Together, these signals reflect a growing consensus: societal participation is not merely complementary to, but co-constitutive to evidence-based regulation. Patient organizations and advocacy networks are increasingly recognized as active co-authors of evidentiary value, helping to define what counts as clinically relevant (e.g., the route of drug administration), ethically acceptable, and economically justified. For the OD ecosystem in particular, marked by small trials, heterogenous populations, and high uncertainty, this shift toward inclusive assessment frameworks holds the potential to reshape how regulatory legitimacy and therapeutic value are determined across Europe.

### 4.4 Ethical and political tensions

While societal participation is often valued for its democratizing potential, this review also highlights underlying tensions and risks. Instrumentalization by commercial sponsors, through selective inclusion of patient voices or strategic use of testimonials, remains a credible threat to the integrity of participatory processes (20). Similarly, unequal representation within and across advocacy groups may reproduce, rather than mitigate, pre-existing health inequities (24).

These risks are further compounded when patient organizations are invited to participate without adequate resources, institutional support, or clearly defined mandates. In such cases, engagement may drift toward tokenism, reinforcing the perception of legitimacy without conferring actual influence.

These observations underscore the need for safeguards: clear governance frameworks, transparency in patient–industry relationships, and mechanisms to ensure inclusivity and legitimacy. Ethically co-designed protocols, independent patient advisory boards, and capacity-building initiatives can all serve to balance power asymmetries and institutionalize participatory integrity (8, 25).

### 4.5 Gaps and research priorities

Despite increasing documentation of societal participation, the field remains empirically underdeveloped in key areas. Most studies are descriptive or cross-sectional, lacking longitudinal data or robust evaluation metrics. For instance, while patient engagement is often associated with improved recruitment or trial design, these claims are rarely substantiated with comparative or counterfactual analyses (13, 29).

There is also limited evidence on how participatory models impact long-term access, pricing negotiations, or regulatory decision-making processes. Patient-generated evidence, including real-world data and preference studies, is often collected outside formal pipelines and remains poorly integrated into structured evaluation frameworks (21).

Emerging work in preference elicitation highlights the potential of systematic approaches to capture what matters most to patients (28). However, more research is needed to understand how these insights can be translated into outcome selection and HTA processes with minimal bias and maximal inclusivity. This includes ensuring that diverse patient populations, not only those organized in well-resourced groups, are meaningfully represented in defining value, benefit, and clinical relevance.

Future research should therefore aim to assess the long-term impact of societal engagement on innovation outcomes, equity, and health system performance, and explore underrepresented geographies and disease areas. Additionally, methodological advances are needed to support the fair translation of patient input into evaluative frameworks that can withstand scientific and regulatory scrutiny. Further work is also needed to examine how advocacy groups’ interpretative contributions, such as those mediating between HTA outputs and patient communities, can be systematically integrated without compromising independence or transparency (26).

### 4.6 Limitations

This review has several limitations that warrant acknowledgment. First, the systematic search was conducted in two major databases (PubMed and Scopus), which may have resulted in the omission of relevant studies indexed elsewhere. Second, despite applying predefined eligibility criteria, the included literature is geographically skewed toward North America and Western Europe, and disease representation is dominated by neuromuscular disorders, particularly SMA. This concentration reflects where societal participation in orphan drug development has been most visible, but it limits the generalizability of the findings to other world regions and disease areas.

Third, the methodological quality of the included studies was heterogeneous. While empirical studies with structured data collection were generally robust, several contributions relied on descriptive accounts, case reports, or policy commentaries without empirical validation. These sources were retained for their conceptual relevance, but their inclusion may introduce interpretive bias.

Finally, most available studies are cross-sectional or descriptive, providing limited evidence on longitudinal impacts or causal relationships between societal participation and drug development outcomes. As a result, while this review maps the breadth of societal contributions, further research is required to establish their long-term effectiveness, equity implications, and integration into regulatory and health technology assessment frameworks.

## 5 Conclusion

This systematic review highlights the evolving role of societal actors in the development of orphan drugs, particularly within the field of neuromuscular and neurodegenerative disorders. Far from being peripheral or symbolic, their contributions span the full drug development lifecycle, acting as Initiators, Accelerators, Translators, and Monitors of innovation. These roles challenge conventional boundaries between scientific, regulatory, and civil domains, and call for frameworks that recognize the epistemic, methodological, and political legitimacy of patient-driven expertise.

As regulatory paradigms evolve, particularly with the implementation of the EU HTA Regulation and the increasing formalization of patient involvement in PICOS-based assessments, the integration of societal perspectives is no longer a matter of consultation, but one of co-production. Preference elicitation studies, co-designed readiness infrastructures, and evolving advisory models illustrate this transition.

Yet important tensions remain: risks of instrumentalization, unequal representation, methodological inconsistency, and insufficient governance clarity continue to limit the effectiveness and inclusivity of these efforts.

Future research and policy must move beyond descriptive accounts toward comparative and evaluative approaches that can assess the impact of participatory models over time. Ensuring that engagement mechanisms are inclusive, transparent, and sustainable will be essential to realizing the full potential of collaborative innovation in rare disease treatment development. Ultimately, the trajectories examined here suggest that patient and advocacy groups are no longer exceptional contributors but structural co-architects of the orphan drug ecosystem, embodying a shift toward genuine patient empowerment, particularly in ultra-rare diseases where traditional innovation pathways have historically failed.

## Data Availability

The original contributions presented in the study are included in the article/Supplementary material, further inquiries can be directed to the corresponding author.

## References

[1] Haffner ME. Adopting orphan drugs — two dozen years of treating rare diseases. N Engl J Med. (2006) 354:445–7. doi: 10.1056/NEJMp058317, PMID: 16452556

[2] Tambuyzer E Vandendriessche B Austin CP Brooks PJ Larsson K Miller Needleman KI . Therapies for rare diseases: therapeutic modalities, progress and challenges ahead. Nat Rev Drug Discov. (2020) 19:93–111. doi: 10.1038/s41573-019-0049-9, PMID: 31836861

[3] Coté TR Xu K Pariser AR. Accelerating orphan drug development. Nat Rev Drug Discov. (2010) 9:901–2. doi: 10.1038/nrd3340, PMID: 21119719

[4] EMA (E.M.A.). (2025). European Medicines Agency (EMA). Research and Development. Human Regulatory Overview. Available online at: https://www.ema.europa.eu/en/human-regulatory-overview/research-development (Accessed on April 30, 2025).

[5] Mavris M Furia Helms A Bere N. Engaging patients in medicines regulation: a tale of two agencies. Nat Rev Drug Discov. (2019) 18:885–6. doi: 10.1038/d41573-019-00164-y, PMID: 31780842

[6] Morel T Arickx F Befrits G Siviero P van der Meijden C Xoxi E . Reconciling uncertainty of costs and outcomes with the need for access to orphan medicinal products: a comparative study of managed entry agreements across seven European countries. Orphanet J Rare Dis. (2013) 8:198. doi: 10.1186/1750-1172-8-198, PMID: 24365263 PMC3882782

[7] de Vrueh RLA de Vlieger JSB Crommelin DJA. Editorial: public-private partnerships as drivers of innovation in healthcare. Front Med (Lausanne). (2019) 6:114. doi: 10.3389/fmed.2019.00114, PMID: 31214590 PMC6554417

[8] Aartsma-Rus A Vroom E O'Reilly D. The role of patient involvement when developing therapies. Nucleic Acid Ther. (2022) 32:118–22. doi: 10.1089/nat.2021.0048, PMID: 34597188 PMC9058870

[9] Bertini E Dessaud E Mercuri E Muntoni F Kirschner J Reid C . Safety and efficacy of olesoxime in patients with type 2 or non-ambulatory type 3 spinal muscular atrophy: a randomised, double-blind, placebo-controlled phase 2 trial. Lancet Neurol. (2017) 16:513–22. doi: 10.1016/S1474-4422(17)30085-6, PMID: 28460889

[10] Gaillard S Roche L Deschênes G Morin D Vianey-Saban C Acquaviva-Bourdain C . Collaboration between academics, small pharmaceutical company and patient organizations in the development of a new formulation of cysteamine in nephropathic cystinosis: a successful story. Therapie. (2020) 75:169–73. doi: 10.1016/j.therap.2020.02.008, PMID: 32248985

[11] Furlong P Bridges JFP Charnas L Fallon JR Fischer R Flanigan KM . How a patient advocacy group developed the first proposed draft guidance document for industry for submission to the U.S. Food and Drug Administration. Orphanet J Rare Dis. (2015) 10:82. doi: 10.1186/s13023-015-0281-2, PMID: 26104810 PMC4486430

[12] Reichel M Murauer EM Steiner M Coch C Trübel H. Philanthropic drug development: understanding its importance, mechanisms, and future prospects. Drug Discov Today. (2025) 30:104298. doi: 10.1016/j.drudis.2025.104298, PMID: 39848487

[13] Nguyen CQ Alba-Concepcion K Palmer EE Scully JL Millis N Farrar MA. The involvement of rare disease patient organisations in therapeutic innovation across rare paediatric neurological conditions: a narrative review. Orphanet J Rare Dis. (2022) 17:167. doi: 10.1186/s13023-022-02317-6, PMID: 35436886 PMC9014615

[14] Epps C Bax R Croker A Green D Gropman A Klein AV . Global regulatory and public health initiatives to advance Pediatric drug development for rare diseases. Ther Innov Regul Sci. (2022) 56:964–75. doi: 10.1007/s43441-022-00409-w, PMID: 35471559 PMC9040360

[15] Peterson I Cruz R Sarr F Stanley AM Jarecki J. The SMA clinical trial readiness program: creation and evaluation of a program to enhance SMA trial readiness in the United States. Orphanet J Rare Dis. (2020) 15:118. doi: 10.1186/s13023-020-01387-8, PMID: 32443972 PMC7564894

[16] Tizzano EF Christie-Brown V Baranello G Germanenko O Gray A Krstic M . Clinical trial readiness for spinal muscular atrophy: experience of an international educational-training initiative. J Neuromuscul Dis. (2022) 9:809–20. doi: 10.3233/JND-221538, PMID: 36314215

[17] Furlong P Dugar A White M. Patient engagement in clinical trial design for rare neuromuscular disorders: impact on the DELIVER and ACHIEVE clinical trials. Res Involvem Engag. (2024) 10:1. doi: 10.1186/s40900-023-00535-1, PMID: 38167117 PMC10759564

[18] Peay HL Biesecker BB Wilfond BS Jarecki J Umstead KL Escolar DM . Barriers and facilitators to clinical trial participation among parents of children with pediatric neuromuscular disorders. Clin Trials. (2018) 15:139–48. doi: 10.1177/1740774517751118, PMID: 29475375 PMC5891354

[19] Bird N Knox L Palmer A Heenen D Blanc P Scobie N . When innovation and commercialization collide: a patient advocate view in neuroblastoma. J Clin Oncol. (2022) 40:120–6. doi: 10.1200/JCO.21.01916, PMID: 34793201

[20] Patterson AM. Emerging roles and opportunities for rare disease patient advocacy groups. Ther Adv Rare Dis. (2023) 4:26330040231164425. doi: 10.1177/26330040231164425 37197559 PMC10184204

[21] Wicks P Vaughan TE Massagli MP Heywood J. Accelerated clinical discovery using self-reported patient data collected online and a patient-matching algorithm. Nat Biotechnol. (2011) 29:411–4. doi: 10.1038/nbt.1837, PMID: 21516084

[22] Mavris M Le Cam Y. Involvement of patient organisations in research and development of orphan drugs for rare diseases in europe. Mol Syndromol. (2012) 3:237–43. doi: 10.1159/000342758, PMID: 23293582 PMC3531929

[23] Huml RA Dawson J Bailey M Nakas N Williams J Kolochavina M . Accelerating rare disease drug development: lessons learned from muscular dystrophy patient advocacy groups. Ther Innov Regul Sci. (2021) 55:370–7. doi: 10.1007/s43441-020-00221-4, PMID: 32974874 PMC7513900

[24] Frost J Hall A Taylor E Lines S Mandizha J Pope C. How do patients and other members of the public engage with the orphan drug development? A narrative qualitative synthesis. Orphanet J Rare Dis. (2023) 18:84. doi: 10.1186/s13023-023-02682-w, PMID: 37069597 PMC10108537

[25] Stein S Bogard E Boice N Fernandez V Field T Gilstrap A . Principles for interactions with biopharmaceutical companies: the development of guidelines for patient advocacy organizations in the field of rare diseases. Orphanet J Rare Dis. (2018) 13:18. doi: 10.1186/s13023-018-0761-2, PMID: 29357903 PMC5778794

[26] Pickaert A-P. Patient involvement in health technology assessments: lessons for EU joint clinical assessments. J Market Access; Health Policy. (2025) 13:38. doi: 10.3390/jmahp13030038 PMC12372039 40860957

[27] Daban M Lacroix C Micallef J. Patients' organizations in rare diseases and involvement in drug information: illustrations with LMC France, the French Association of Chronic Myeloid leukemia. Therapie. (2020) 75:221–4. doi: 10.1016/j.therap.2020.02.014, PMID: 32113687

[28] Jimenez-Moreno AC van Overbeeke E Pinto CA Smith I Sharpe J Ormrod J . Patient preferences in rare diseases: a qualitative study in neuromuscular disorders to inform a quantitative preference study. Patient. (2021) 14:601–12. doi: 10.1007/s40271-020-00482-z, PMID: 33660162 PMC8357717

[29] Gusset N Stalens C Stumpe E Klouvi L Mejat A Ouillade MC . Understanding European patient expectations towards current therapeutic development in spinal muscular atrophy. Neuromuscul Disord. (2021) 31:419–30. doi: 10.1016/j.nmd.2021.01.012, PMID: 33752935

[30] van Overbeeke E Janssens R Whichello C Schölin Bywall K Sharpe J Nikolenko N . Design, conduct, and use of patient preference studies in the medical product life cycle: a multi-method study. Front Pharmacol. (2019) 10:1395. doi: 10.3389/fphar.2019.01395, PMID: 31849657 PMC6902285

