# Supplementary material for: Societal participation in the development of orphan drugs: a systematic review
Source: Front Med (Lausanne). 2025 Sep 10;12:1653304. doi: 10.3389/fmed.2025.1653304 (PMC12457289; doi:10.3389/fmed.2025.1653304)
Supplement: Supplementary file 1 [file Table_1.docx]

Supplementary Table S1. Summary of Societal Participation by Role Type

| **Role** | **Definition** | **Examples from Included Studies** | **Key Outcomes** |
| --- | --- | --- | --- |
| **Initiator** | Launching or resuscitating drug development programs in the absence of sufficient commercial or academic interest. | AFM-Téléthon funding of Olesoxime trials (Bertini et al., 2017); Philanthropic-led initiatives in neglected rare diseases (Reichel et al., 2025); Patient-led online trial in ALS (Wicks et al., 2011). | Early-stage risk-sharing; Triggering R&D where no commercial incentive existed; Mobilization of dispersed expertise. |
| **Accelerator** | Reducing bottlenecks in trial conduct through logistical, educational, or infrastructural support. | Trial-readiness platforms by Cure SMA (Peterson et al., 2020); Pre-consent materials and trial logistics co-designed with families in DMD (Furlong et al., 2024); Educational initiatives for trial quality (Tizzano et al., 2022). | Improved trial recruitment and retention; Reduced administrative delays; Enhanced preparedness of families and clinicians. |
| **Translator** | Aligning research design and interpretation with patient-relevant endpoints and lived experience. | Patient preference studies in neuromuscular disorders (Jimenez-Moreno et al., 2021); Families shaping endpoint selection in SMA/DMD (Peay et al., 2018; Gusset et al., 2021); EMA guidance co-developed with PPMD (Furlong et al., 2015). | Increased endpoint relevance; Validation of outcome measures; Greater acceptability and ethical robustness of trials. |
| **Monitor** | Ensuring ethical governance, accountability, and equitable access through advocacy and oversight. | PAG–pharma interaction guidelines (Stein et al., 2018); Advocacy for transparent reimbursement and HTA (Pickaert, 2025); Watchdog coalitions lobbying for access equity (Patterson et al., 2023). | Strengthened legitimacy of development; Promotion of transparency; Accountability in regulatory and market access processes. |
