# Supplementary material for: Societal participation in the development of orphan drugs: a systematic review
Source: Front Med (Lausanne). 2025 Sep 10;12:1653304. doi: 10.3389/fmed.2025.1653304 (PMC12457289; doi:10.3389/fmed.2025.1653304)
Supplement: Supplementary file 2 [file Data_Sheet_1.docx]

APPENDIX A . PRISMA 2020 Flow Diagram of the Literature Screening and Inclusion Process

**Identification of studies via databases and registers**

Records removed *before screening*:

Duplicate records removed (n = 10 )

Records marked as ineligible by automation tools (n = 0 )

Records removed for other reasons (n = 0 )

Records identified from:

Databases (n =94 (70+24*) )

Registers (n =0 )

**Identification**

Records screened

(n = 84) (94 - 10 doublons)

Records excluded**

(n = 60 )

Reports sought for retrieval

(n = 24)

Reports not retrieved

(n = 0 )

**Screening**

Reports assessed for eligibility

(n = 24)

Reports excluded:

Reason 1 (n = 2)**

Studies included in review

(n = 22)

**Included**

* Additional records identified through manual search (e.g., Google Scholar, Orphanet, expert recommendations). Twenty records were screened; seven met the eligibility criteria and were included in the final synthesis.

** Exclusion criteria applied during full-text screening: absence of empirical data, non-neuromuscular or non-neurodegenerative focus, review or opinion articles, or lack of documented societal participation.

Source: Page MJ, et al. BMJ 2021;372:n71. doi: 10.1136/bmj.n71.

This work is licensed under CC BY 4.0. To view a copy of this license, visit <https://creativecommons.org/licenses/by/4.0/>
