# Supplementary material for: Societal participation in the development of orphan drugs: a systematic review
Source: Front Med (Lausanne). 2025 Sep 10;12:1653304. doi: 10.3389/fmed.2025.1653304 (PMC12457289; doi:10.3389/fmed.2025.1653304)
Supplement: Supplementary file 3 [file Data_Sheet_2.docx]

APPENDIX B. Quality Assessment of Included Studies: Appraisal Tools, Ratings, and Limitations

| **Reference** | **Study Type** | **Tool Used** | **Quality Rating** | **Key Limitations** |
| --- | --- | --- | --- | --- |
| **Furlong et al. (2015)** | Qualitative case study | JBI Checklist (Case Reports) | High | Limited generalisability but strong reflexivity and partner integration |
| **Furlong et al. (2024)** | Qualitative descriptive | JBI Checklist (Qualitative) | Moderate | Good practical insights, less structured methodological transparency |
| **Mavris & Le Cam (2012)** | Policy report with stakeholder consultation | JBI Checklist (Expert Opinion) | High | Strong policy relevance but lacks reproducibility of stakeholder input methods |
| **Moreno et al. (2021)** | Qualitative interviews | CASP Qualitative Checklist | High | Well-conducted with thematic saturation but lacks triangulation |
| **Wicks et al. (2011)** | Online observational cohort | ROBINS-I | Moderate | Innovative digital recruitment but high risk of self-selection bias |
| **Frost et al. (2023)** | Narrative synthesis | CASP Qualitative Checklist | Moderate | Rich synthesis but lacks methodological transparency and participant role definition |
| **Peay et al. (2014)** | Qualitative survey study | JBI Checklist (Qualitative) | Moderate | Sociologically grounded, limited by survey tool standardisation |
| **Reichel et al. (2020)** | Stakeholder analysis | CASP (Stakeholder Engagement) | High | Robust mapping, limited depth of participant validation |
| **Bird et al. (2021)** | Patient-led perspective article | JBI Checklist (Viewpoint) | Moderate | Rich perspective but not empirical; lacks systematic structure |
| **Peterson et al. (2020)** | Cross-sectional survey | AXIS | Moderate | Clear structure, but sampling bias and lack of tool validation |
| **Tizzano et al. (2022)** | Mixed-method implementation study | ROBINS-I | Moderate | Well-executed implementation model; lacks standardised outcome measures |
| **Stein et al. (2018)** | Qualitative content analysis | CASP (Qualitative synthesis) | Moderate | Solid structure; limited reproducibility |
| **Patterson et al. (2023)** | Narrative synthesis | CASP | High | Synthesis clear and well framed; lacking diversity of contexts |
| **Gaillard et al. (2019)** | Case study | JBI Checklist (Case Report) | Moderate | Case well described; lacks external validation |
| **Bertini et al. (2017)** | Empirical case report | JBI Checklist (Case Report) | High | Contextually rich, but single-case limitation |
| **Pickaert (2025)** | Policy analysis with stakeholder input | JBI Checklist (Qualitative/Expert Opinion) | High | Provides structured insights into EU HTA patient involvement; limited by reliance on stakeholder narratives |
| **Nguyen et al. (2018)** | Policy mapping | Not applicable (mapping) | Not rated | No empirical data; descriptive policy scope |
| **Gusset et al. (2021)** | Position paper | Not formally appraised (non-empirical) | Not applicable | No empirical component; opinion-based synthesis only |
| **Daban et al. (2024)** | Policy commentary | Not applicable | Not rated | Commentary format; not structured for appraisal |
| **Aartsma-Rus et al. (2022)** | Perspective | Not applicable (commentary) | Not rated | Expert opinion; not suitable for formal quality rating |
| **Huml et al. (2021)** | Expert commentary | Not applicable (commentary) | Not rated | Non-empirical policy analysis |
| **Epps et al. (2021)** | Narrative commentary | Not applicable (commentary) | Not rated | Discussion paper; not formally appraised |
